# Neural correlates of auditory temporal predictions during sensorimotor synchronization

**DOI:** 10.3389/fnhum.2013.00380

**Published:** 2013-08-21

**Authors:** Nadine Pecenka, Annerose Engel, Peter E. Keller

**Affiliations:** ^1^Music Cognition and Action Group, Max Planck Institute for Human Cognitive and Brain SciencesLeipzig, Germany; ^2^Cognitive and Behavioral Neuroscience Unit, D'Or Institute for Research and EducationRio de Janeiro, Brazil; ^3^Music Cognition and Action Group, The MARCS Institute, University of Western SydneySydney, NSW, Australia

**Keywords:** temporal prediction, sensorimotor synchronization, medial prefrontal cortex, motor timing, dual-task interference

## Abstract

Musical ensemble performance requires temporally precise interpersonal action coordination. To play in synchrony, ensemble musicians presumably rely on anticipatory mechanisms that enable them to predict the timing of sounds produced by co-performers. Previous studies have shown that individuals differ in their ability to predict upcoming tempo changes in paced finger-tapping tasks (indexed by cross-correlations between tap timing and pacing events) and that the degree of such prediction influences the accuracy of sensorimotor synchronization (SMS) and interpersonal coordination in dyadic tapping tasks. The current functional magnetic resonance imaging study investigated the neural correlates of auditory temporal predictions during SMS in a within-subject design. Hemodynamic responses were recorded from 18 musicians while they tapped in synchrony with auditory sequences containing gradual tempo changes under conditions of varying cognitive load (achieved by a simultaneous visual *n*-back working-memory task comprising three levels of difficulty: observation only, 1-back, and 2-back object comparisons). Prediction ability during SMS decreased with increasing cognitive load. Results of a parametric analysis revealed that the generation of auditory temporal predictions during SMS recruits (1) a distributed network of cortico-cerebellar motor-related brain areas (left dorsal premotor and motor cortex, right lateral cerebellum, SMA proper and bilateral inferior parietal cortex) and (2) medial cortical areas (medial prefrontal cortex, posterior cingulate cortex). While the first network is presumably involved in basic sensory prediction, sensorimotor integration, motor timing, and temporal adaptation, activation in the second set of areas may be related to higher-level social-cognitive processes elicited during action coordination with auditory signals that resemble music performed by human agents.

## Introduction

The ability to predict the time course of events as they unfold in the immediate environment is a fundamental skill that underlies most activities in everyday life. This ability is paramount in situations that require the coordination of our actions in time and space with the actions of other people around us. Highly refined forms of such interpersonal action coordination can be found in the music domain, where action synchronization between performers is often required under challenging circumstances. For instance, in Western classical music, musical expression entails the introduction of local tempo deviations in order to communicate particular interpretations and to convey emotions to the audience (e.g., Palmer, 1997; Clarke, 1999; Gabrielsson and Lindström, 2001; Chaffin and Logan, 2006). Nevertheless, interpersonal action coordination in musical ensembles can still be extremely accurate. Studies of small ensembles with two or three instruments report that sounds that are notated to be produced simultaneously by different instrumental voices are typically played with small asynchronies between them, with a spread of only around 30–50 ms or less (Rasch, 1979, 1988; Shaffer, 1984; Palmer, 1997; Goebl and Palmer, 2009; Keller and Appel, 2010). Furthermore, this high level of synchrony can be retained during expressive musical passages that are characterized by considerable deviations from isochronous tempo (Shaffer, 1984). Such a high degree of temporal precision during interpersonal synchronization cannot be achieved if musicians only react to the perceived actions of their co-performers. Musicians presumably rely on anticipatory mechanisms, among other processes, which allow them to predict the sounds that will be produced by their co-performers and coordinate their own anticipated actions with these predictions (see Keller, 2008).

Paced finger-tapping tasks are one of the most common paradigms used to investigate basic musical SMS processes in an experimental setting. In these tasks, individuals are normally asked to tap a finger in time with an auditorily presented pacing signal, while the asynchronies between taps and pacing events are analyzed to yield different measures of individual SMS performance (for an overview, see Repp, 2005, 2006; Repp and Su, 2013). The auditory pacing signals can be regular (i.e., metronomic) or irregular, e.g., include local timing perturbations or gradual tempo changes as found in performed classical music.

Repp (2002) proposed two correlational measures as indices of synchronization ability in tasks requiring finger tapping to expressive music. These measures are derived by computing the cross-correlation (CC) between an individual's inter-tap intervals (ITIs) and the inter-onset intervals (IOIs) between tones in the musical sequence at two different lags: lag-0 and lag-1. If an individual is able to predict upcoming tempo modulations in the auditory sequence, relatively high lag-0 CCs reflect the fact that he or she adjusted their ITIs more strongly on the basis of *upcoming* rather than preceding IOIs in the sequence. Conversely, relatively high lag-1 CCs reveal that the individual tended to react to rather than predict the pattern of tempo changes in the IOIs, i.e., the individual adjusted his or her tap timing more strongly on the basis of *preceding* rather than upcoming IOIs in the auditory sequence. Tracking behavior has been mainly observed for SMS with random or barely detectable timing modulations (e.g., Michon, 1967; Thaut et al., 1998, 2009; Stephan et al., 2002; Madison and Merker, 2005; Schulze et al., 2005), or when participants were unaware that they were tapping along with a pacing sequence that mirrored the expressive timing profile of a complex musical piece (Repp, 2002, 2006). On the other hand, when timing variations in the auditory sequence are easily detectable and follow a regular or familiar pattern (e.g., the local tempo variations of a well-known musical piece), participants are able to anticipate these changes (e.g., Michon, 1967; Repp, 2005; Rankin et al., 2009) and, furthermore, their performance improves with training (Repp, 2002).

Our own previous research has focused on anticipation during SMS by investigating temporal prediction abilities in tasks requiring individuals to tap a finger in synchrony with a tempo-changing pacing signal (Pecenka and Keller, 2009a,b, 2011). In these studies, gradual tempo transitions were designed to resemble tempo variations found in real performed music (i.e., accelerando and ritardando). To assess the degree to which individuals predicted upcoming tempo changes, we computed the ratio of the lag-0 over the lag-1 CCs between the individual's ITIs and the IOIs of the pacing signal (cf. Repp, 2002). This prediction/tracking ratio reflects whether individuals show relatively stronger prediction (ratio > 1) or tracking (ratio < 1) tendencies during SMS with ongoing tempo changes. The first two studies (Pecenka and Keller, 2009a,b) revealed that about two-thirds of the individuals in our samples (comprised mostly of amateur musicians) tended to predict the timing variations, while the remaining individuals displayed a relatively stronger tendency to track the ongoing tempo changes. Prediction/tracking ratios were approximately normally distributed and positively correlated with SMS ability (see Pecenka and Keller, 2009a,b). In a third study (Pecenka and Keller, 2011) we found that the individual differences in prediction ability impacted upon interpersonal action coordination in a joint finger-tapping task. Furthermore, individual differences in prediction ability, which were in those settings highly reliable and stable across time were positively correlated with musical experience and self-reported tendency to take other performers' perspectives during musical interactions (Pecenka and Keller, 2011).

Although temporal prediction is inherent in practically any kind of SMS task, the underlying mechanisms and brain processes of temporal prediction during SMS have not yet been investigated. Keller (2008) has proposed that musicians make predictions about their co-performers' ongoing action outcomes by using internal simulation processes to generate anticipatory auditory images of the others' sounds. This assumption is supported by findings from our previous studies, which revealed a positive correlation between prediction/tracking ratios and the acuity of auditory imagery for pitch (Pecenka and Keller, 2009a) and timing (Pecenka and Keller, 2009b). Empirical and theoretical work has furthermore suggested that the formation of auditory images relies (although not solely) on working memory (Baddeley and Logie, 1992; Smith et al., 1995; see Hubbard, 2010 for a review), and corresponding brain areas have been found to be active during auditory imagery (Aleman and van't Wout, 2004).

We hypothesized that introducing a simultaneous working-memory task during finger tapping with tempo-changing pacing sequences would lead to variations in the individual degree of prediction to the extent that both tasks draw on similar working-memory processes. A pilot study conducted beforehand supported this hypothesis and indicated that intraindividual prediction ability decreases with increasing cognitive load imposed by simultaneous working-memory computations^1^. The aim of the current study was to identify patterns of neural activation that covary as a function of prediction tendencies during synchronized finger tapping. To this end, we employed functional magnetic resonance imaging (fMRI) to investigate the neural correlates of temporal prediction during SMS. We have used a within-subjects experimental design (rather than a between-subjects correlational approach based on individual differences), as it was possible to manipulate individual prediction/tracking ratios experimentally. Participants were asked to tap a finger in synchrony with an auditory pacing signal that changed its tempo gradually. Simultaneously, cognitive load was varied by means of a visual *n*-back working-memory task with three levels of increasing difficulty: object observation, 1-back, and 2-back object comparisons. According to our pilot study, we expected that the degree of temporal prediction measured during finger tapping will decrease with increasing visual working-memory load. A parametric analysis of the hemodynamic responses was conducted to reveal activations in brain networks that covary with the degree of temporal prediction measured during synchronized finger tapping with a tempo-changing pacing signal.

## Materials and methods

### Participants

Twenty musicians (10 females) participated in the study. Two participants were excluded from the analyses due to incomplete behavioral data and excessive head movement during fMRI data acquisition. The mean age of the remaining sample of 18 participants (9 females) was 25.5 years (*SD* = 3.79). Participants were amateur musicians (*n* = 9) and university music students (*n* = 9) with varying amounts of musical experience (years of instrument playing/singing summed over all instruments: range = 11–57, *M* = 27.7, *SD* = 13.1). Participants had started playing an instrument (or singing) when aged between 4 and 16 years (*M* = 7.8, *SD* = 2.9) and played one to four instruments (*M* = 2.8, *SD* = 1.1). All participants were right-handed according to the Edinburgh handedness inventory (Oldfield, 1971) and had no history of psychiatric, major medical, or neurological disorder. After being informed about potential risks of participation in an fMRI study and being screened for exclusion criteria by a physician at the institute, participants gave their written informed consent to undertake the study. The experiment was performed in accordance with ethical standards compliant with the Declaration of Helsinki and approved by the ethics committee of the University of Leipzig.

### Stimuli

#### Auditory stimuli

**Figure 1:**
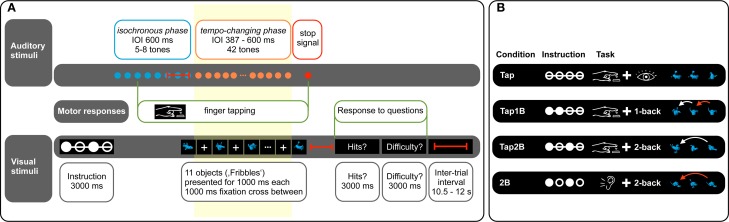
**(Panel A)** provides an overview over auditory and visual stimuli presented in the experiment. Finger tapping is only performed in the tapping conditions (Tap, Tap1B, Tap2B). The question regarding *n*-back hits (“Hits?”) is only presented in the *n*-back conditions (Tap1B, Tap2B, 2B). Red lines indicate temporal jitter implemented to decorrelate predictors of the fMRI design. Hemodynamic responses recorded during the tempo-changing phase (yellow box) were used to model the predictors of interest in the fMRI analyses. **(Panel B)** displays the experimental conditions of the dual task. Task instruction was given symbolically by a constellation of filled and empty circles (indicating the visual *n*-back task requirement) and a line connecting these circles (informing about a finger-tapping condition). A selection of novel objects (“Fribbles”) is presented as an example. Red arrows indicate hits in the visual *n*-back task.

During each trial, 47–50 identical pacing sounds (1000 Hz sine-wave tones with 200 ms duration and 10 ms linear rise and decay ramps) were presented (to guide finger tapping in experimental conditions 1–3, see Figure 1A). The number of initial pacing sounds was varied from 5–8 tones in order to implement a variable jitter in the time point at which the tempo changes started. These initial pacing sounds (*isochronous phase*) were presented with an IOI of 600 ms, after which the tempo changed gradually (*tempo-changing phase*). Tempo changes were designed to resemble tempo variations found in performed music (accelerando and ritardando) and followed quadratic functions. Each trial contained 6 tempo changes that proceeded over 5, 8, or 10 pacing tones (corresponding to 5, 7, or 11% change rate, respectively) and spanned a tempo range from 387 to 600 ms IOI. The end of each trial was signaled by an auditory stop signal (800 Hz sine-wave tone with 200 ms duration and 10 ms linear rise and decay ramp). Overall 14 trials with different pacing sequence versions were presented randomly intermixed, with each version being presented once per experimental condition (see for a sound example Audio S1 in the Supplementary Audio Material).

#### Visual stimuli

In order to implement a visual distractor task, a set of 11 novel abstract objects (from a catalog of 36 objects, called “Fribbles”, Williams, 1998; obtained with permission from the Tarr lab website: http://tarrlab.cnbc.cmu.edu/; see Figure 1B) was presented sequentially during each trial. The visual objects were displayed centrally in blue on a black background, and presented one after another for a duration of 1000 ms with an intervening white fixation cross (duration 1000 ms, displayed at the center of the screen; see Figure 1A). Written task instructions were presented only at the beginning of the experiment. Prior to each trial, task conditions were coded symbolically: a constellation of filled and empty circles symbolized the *n*-back memory task requirement, while the presence of a line connecting these circles informed the participant about a tapping vs. no-tapping condition. This visual task instruction was displayed for 3000 ms at the beginning of each trial. We have used a non-verbal dual task in order to avoid covert subvocalization, which is assumed to activate motor regions [such as the supplementary motor area (SMA); e.g., Halpern et al., 2004], to reduce the likelihood of overlap with motor regions activated by the finger-tapping task.

### Procedure

The experiment included a total of four experimental conditions (see Figure 1B): (1) Finger tapping and object observation (Tap), (2) finger tapping and 1-back object comparisons (Tap1B), (3) finger tapping and 2-back object comparisons (Tap2B), and (4) 2-back object comparisons without finger tapping (2B). At the beginning of each trial, participants were symbolically instructed which of the experimental conditions they would encounter.

Auditory stimulation began after the visual instruction had been on screen for 3 seconds (see Figure 1A). In finger-tapping conditions (Tap, Tap1B, Tap2B), participants were asked to tap their right index finger on a custom-built MRI-compatible tapping pad in synchrony with the pacing signal, starting with the third pacing sound. (Condition 2B served as a control condition, where participants only performed the 2-back working-memory task while receiving auditory stimulation without finger-tapping requirement). The tapping pad had dimensions 210 mm long, 150 mm wide, and 25 mm deep, with a 70 × 70 mm area on its upper surface marked as the target for finger taps. Taps on the pad were registered by an air-pressure sensor, which emitted a trigger that was sent to the computer running the experiment. Depending on jittering, visual object presentation started with the 5th, 6th, 7th, or 8th pacing tone, which coincided with the last regular tone of the isochronous phase. In the tapping only condition (Tap), participants were required to attend visually to the presented objects. In the remaining conditions, individuals were asked to either perform 1-back (Tap1B) or 2-back object identity comparisons (Tap2B and 2B), while keeping track of the total number of *n*-back hits in each trial. The end of auditory and visual object presentation was signaled by the auditory stop signal in each trial.

After a variable time interval (0–1500 ms, jittered in steps of 500 ms), participants were asked to enter the responses to either one (Tap) or two subsequently presented questions (Tap1B, Tap2B, 2B) by moving a cursor (left or right) on a horizontal 5-point visual rating scale. In conditions where *n*-back comparisons were performed (Tap1B, Tap2B, 2B), participants were visually instructed to report the number of *n*-back hits (prompted by the German equivalent for “hits?”) in the foregoing trial, ranging from “1” to “5.” In all four conditions, participants were asked to rate the overall difficulty (prompted by the German equivalent for “difficulty?”) of each trial ranging from “very easy” (1) to “very difficult” (5). All responses were performed with the left hand on a two-button response box and participants were allowed 3 s to complete each question.

Depending on condition and jittering, the total trial length varied between ~30 and 36 s. Trials were separated by a variable inter-trial interval of 10.5–12 s duration (jittered in steps of 500 ms). Trials were pseudo-randomized between conditions. Overall, 56 trials (14 trials per experimental condition) were completed during ~42 min of continuous scanning.

Participants were familiar with the task prior to MRI measurement, as they had performed a near-identical dual task in a behavioral pilot experiment 6 months before scanning (details on the pilot experiment are included in Footnote 1). In addition, detailed verbal and written task instructions were given prior to scanning and participants could practice the task until they felt comfortable with performing it. During the whole experiment, participants lay supine on the scanner bed, with their right hand resting on a custom-built pressure-sensitive MIDI tapping pad. Middle and index finger of the left hand were positioned over two buttons of a response box. Participants were carefully stabilized with form-fitting cushions to prevent arm and head motion. To attenuate scanner noise, participants were provided with earplugs. Auditory stimuli were presented over MRI compatible headphones (MR Confon GmbH, www.mr-confon.de). Visual stimuli and instructions were projected by an LCD projector onto a screen positioned behind the participant's head, which was viewed through a mirror on top of the head coil. Stimulus delivery and response registration was controlled by Presentation 14.7 software (Neurobehavioral Systems, www.neurobs.com) running on a Windows computer. There was a transmission delay of 46 ms between the tapping pad and the registration software, which was subtracted from the recorded tap times before data analysis.

### fMRI data acquisition

Functional imaging was conducted on a 3T scanner (Siemens TRIO, Erlangen, Germany) equipped with the standard birdcage head coil. Thirty-one axial slices (matrix 64 × 64, field of view 192 mm, in plane resolution 3 × 3 mm, slice thickness 3 mm, interslice gap 1 mm) positioned parallel to the bicommisural plane (AC–PC) were acquired using a single-shot gradient echo-planar imaging (EPI) sequence (*TE* = 30 ms flip angle 90°, acquisition bandwidth 116 kHz, repetition time 2000 ms, ascending slice acquisition order). In total, 1265 functional images (volumes) were acquired in a single run.

Geometric distortions were characterized by a B0 field-map scan. The field-map scan consisted of a gradient-echo readout (24 echoes, inter-echo time 0.95 ms) with a standard 2D phase encoding. The B0 field was obtained by a linear fit to the unwrapped phases of all odd echoes.

In addition, a high-resolution whole-brain image (spatial resolution: 1 × 1 × 1.5 mm, *TE* = 3.93 ms, flip angle 10°, *FOV* = 256 × 240 mm, slab thickness = 192 mm, 128 partitions) using a T1-weighted 3D MP-RAGE (magnetization-prepared rapid gradient echo) sequence was acquired for each participant, either in a separate session or directly following the experimental task.

### Behavioral data analysis

#### Finger tapping

In a first computational step in the analysis of SMS data, pacing sounds and corresponding finger taps were aligned by assigning each pacing sound to the closest finger tap within a time window of ±200 ms. Subsequently, three indicators of individual SMS performance were computed: (1) Mean absolute asynchrony (i.e., the unsigned time difference between each finger tap and the corresponding pacing sound) was calculated as an index of tapping *accuracy*, with low asynchronies indicating high SMS accuracy. Furthermore, we calculated (2) the variability of signed asynchronies (i.e., the *SD* of signed within-trial asynchronies) to inform about tapping *precision*, with low variability reflecting high SMS precision. Both measures were computed at the level of single trials, and then averaged across trials within each condition, as well as across conditions, for each participant.

Finally, we computed (3) prediction tendencies based on the lag-0 and lag-1 cross-correlations between the ITIs produced by the participant and the IOIs of the pacing signal. As suggested by Repp (2002), both the prediction index (lag-0 CC) and the tracking index (lag-1 CC) were computed in relation to the lag-1 autocorrelation (AC) of the timing sequence (i.e., CC-AC/1-AC). After this correction, relatively *low* prediction and tracking indices indicate a relatively high degree of prediction and tracking, respectively. To yield a prediction/tracking ratio that is comparable with our previous studies (i.e., ratios > 1 indicate relatively stronger prediction than tracking, while the opposite is true for ratios < 1), it was computed as the ratio of the tracking (lag-1 CC) over prediction (lag-0 CC) index. First, prediction/tracking ratios were computed separately for each trial. In a second step, prediction/tracking ratios were averaged across trials of each condition, as well as across conditions (using Fisher *Z*-scores). In rare cases (i.e., in less than 2% of all trials) where an individual's prediction/tracking ratio in one trial yielded a value exceeding 2.5 *SD* of the individual's mean ratio within a condition, this prediction/tracking ratio was replaced by the mean value of the remaining non-outlying trials of this condition. These corrected prediction/tracking values for separate trials were used for the parametric analysis of fMRI data. In addition, the lag-0 and lag-1 CCs were analyzed separately to investigate whether the prediction and tracking indices were influenced differently by the experimental manipulation. Please note that these absolute values are not informative about an individual's prediction ability. It is only when considered in relation to one another (as a ratio) that they are correlated with SMS performance and reflect the degree to which an individual predicts rather than or tracks (or follows) the ongoing tempo changes in a pacing sequence.

#### Response to questions

Ratings of subjectively perceived task difficulty were averaged across all trials within each experimental condition. To yield an index of working-memory task performance, we computed the deviation of the given response from the correct number of identity hits (1–5) within each trial. These error values were then averaged across trials within each experimental condition.

#### Statistical analyses

Separate analyses of variance (ANOVAs) were run to investigate the global effect of the working-memory manipulation on the degree of prediction (i.e., the prediction/tracking ratio and the separate prediction and tracking indices), SMS performance and task difficulty. When the assumption of sphericity was not met, the Greenhouse-Geisser correction (on the degrees of freedom) was applied. Two-tailed paired *t*-tests were employed to compare individual experimental conditions with each other. The level of significance for these comparisons incorporated a Bonferroni correction, taking the number of tests into account.

### fMRI data analysis

Functional imaging data were analyzed using Statistical Parametric Mapping (SPM8, Wellcome Trust Centre for Neuroimaging, University College London, www.fil.ion.ucl.ac.uk/spm) running under Matlab 7.11 (Mathworks Inc., www.mathworks.com). Images of each participant were realigned to the first image and unwarping was applied. Image distortions were corrected using a B0 field-map scan and images were coregistered to the 3D anatomical image of the participant. The 3D anatomical images were normalized into standard stereotactic space (Montreal Neurological Institute template) and obtained parameters were used for normalization of the functional data. Finally, functional images were spatially smoothed with an 8 mm full-width half-maximum Gaussian filter and high-pass filtered with a cutoff period of 400 s.

Activated voxels were identified by the General Linear Model (GLM). In a first level analysis, two models were computed. Each of these two GLMs comprised predictors of interest that were obtained by modeling a boxcar function with a length of 22 s (corresponding to the length of the tempo-changing phase in a trial, see Figure 1A) convolved with the canonical hemodynamic response function and the temporal derivative. Furthermore, predictors of no interest—task instruction, the isochronous phase, and the responses to the questions in each trial—were included in each of the models.

The primary aim of this study was to reveal brain areas that covary systematically in Blood Oxygen Level Dependent (BOLD) signal amplitude with the degree of prediction during SMS. To this end, a parametric analysis was conducted within a GLM. At the level of each participant, all tapping trials (Tap, Tap1B, Tap2B) were modeled together as a single predictor and weighted with (a) the individual prediction/tracking ratio from each trial as regressor of interest and (b) two further regressors of no interest, i.e., participants' difficulty ratings (values ranging from 1 to 5) and working-memory demands (value 0 for the trials without a working-memory task, condition Tap; value 1 for trials with a 1-back working-memory task, condition Tap1B; value 2 for trials with a 2-back working-memory task, condition Tap2B). In addition, trials in which only the 2-back working-memory task was performed (condition 2B) were modeled as a further predictor. Contrast images for parameters of the prediction/tracking ratio, the condition 2B and the contrast “prediction-2B” were used in second level for random effects analyses (one-sample *t*-test and conjunction analyses).

In a second set of analyses, we investigated the networks of brain areas that were related to the different experimental conditions. Within the first level GLM, four predictors of interest were modeled for each participant, corresponding to the four experimental manipulations: Tap, Tap1B, Tap2B, and 2B. For each participant, individual contrasts were generated for activations related to finger tapping (i.e., Tap2B-2B). These contrast images were entered into a second-level one-sample *t*-test for random effects group analysis.

Furthermore, conjunction analyses (Nichols et al., 2005) were calculated in order to identify shared activations between brain networks activated by working memory (2B) and brain areas positively and negatively related to prediction.

For all second level whole brain group analyses, we only report clusters meeting an extent threshold of *k* > 10 voxels and a significance level of *p* < 0.05 corrected (using False Discovery Rate correction, FDR, for multiple comparisons).

Displays of activations were created by means of the software package MRIcron (www.mccauslandcenter.sc.edu/mricro/mricron/index.html) by superimposing SPM *t*-maps resulting from the second level analyses on a mean anatomy obtained by averaging the normalized anatomy of the 18 analyzed participants. The probabilistic atlas by Diedrichsen et al. (2009) included in the SPM anatomy toolbox Version 1.8 (Eickhoff et al., 2005; Version 1.8 released in 2011) was used to determine cytoarchitectonic probabilities for cerebellar activations.

## Results

### Behavioral data

#### SMS performance

**Figure 2:**
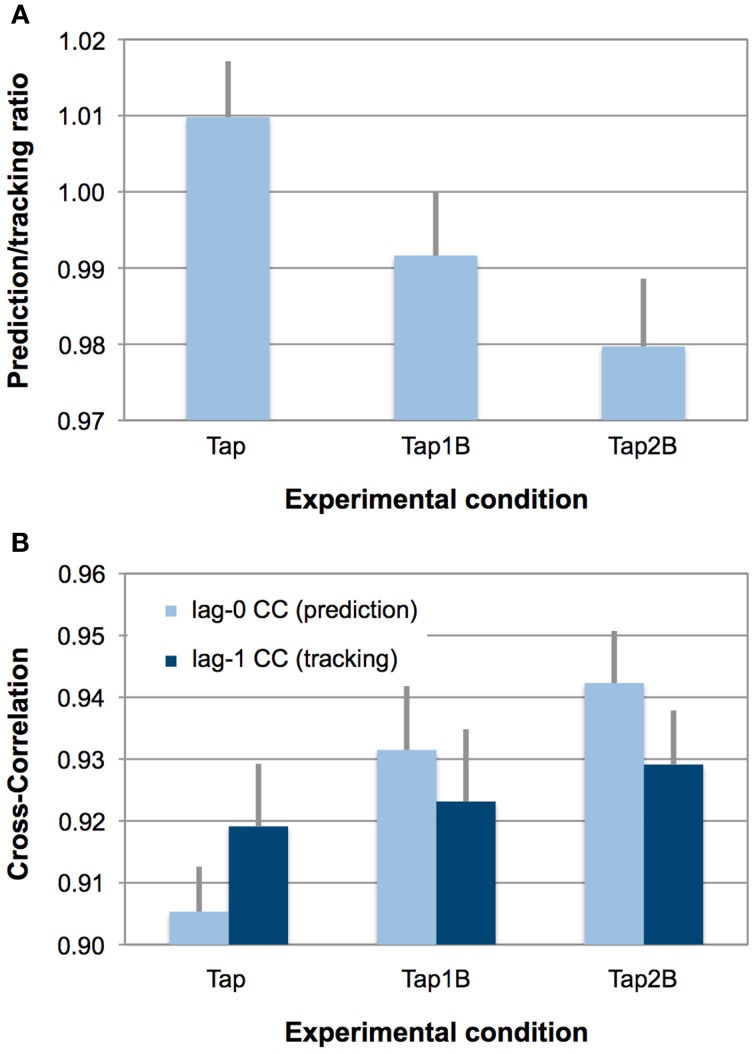
**(Panel A)** displays observed mean prediction/tracking ratios for the three finger-tapping conditions. Error bars display the standard error of means. Prediction/tracking ratios > 1 indicate relatively stronger temporal prediction than temporal tracking during SMS, prediction/tracking ratios < 1 indicate relatively stronger temporal tracking (i.e., weak prediction). **(Panel B)** provides an overview over lag-0 and lag-1 CCs separately for the three finger-tapping conditions. Error bars display the standard error of means. Due to the autocorrelation correction, lower indices indicate stronger prediction and tracking.

An overview over SMS performance, difficulty ratings and *n-back* performance errors in the three finger-tapping conditions is provided in Table 1. Repeated-measures ANOVAs were computed to test whether the experimental manipulation (visual *n*-back task) had the intended effect on the degree of prediction during SMS. The ANOVA yielded a significant main effect of experimental condition on the prediction/tracking ratio [*F*_(2, 34)_ = 16.83, *p* < 0.001, η^2^ = 0.50; see also Figure 2A]. Paired comparisons between individual conditions revealed that prediction/tracking ratios were significantly higher during finger tapping without working-memory demands (Tap) than in the Tap1B [*t*_(17)_ = 4.07, *p* < 0.01] and Tap2B conditions [*t*_(17)_ = 5.01, *p* < 0.001]. Prediction tendencies did, however, not differ significantly between the two working-memory conditions [*t*_(17)_ = 2.34, *p* = 0.03; the significance threshold due to Bonferroni correction is *p* = 0.016].

**Table 1:** **Summary statistics for the four experimental conditions**.

	**Experimental condition**			
	**Tap**	**Tap1B**	**Tap2B**	**2B**
Prediction/tracking ratios	1.01 (0.03)	0.99 (0.04)	0.98 (0.04)	–
Lag-0 CCs (prediction index)	0.91 (0.03)	0.93 (0.04)	0.94 (0.04)	
Lag-1 CCs (tracking index)	0.92 (0.04)	0.92 (0.05)	0.93 (0.04)	
Mean absolute asynchronies (in ms)	52.14 (15.23)	57.50 (13.85)	59.06 (14.48)	–
*SD* of signed asynchronies	47.14 (11.22)	53.61 (13.64)	55.28 (11.71)	–
Perceived task difficulty	1.39 (0.54)	2.31 (0.58)	4.10 (0.50)	3.85 (0.47)
Average number of errors per *n*-back trial	–	0.25 (0.26)	0.68 (0.29)	0.66 (0.24)

Mean scores and standard deviations (in brackets) for the indicators of finger-tapping performance, perceived task difficulty (1 = “very easy”; 5 = “very difficult”), and n-back task performance. Lower values of the lag-0 and lag-1 cross-correlations (CCs) indicate stronger prediction and tracking, respectively. Lower mean absolute asynchronies indicate higher finger-tapping accuracy, while lower SDs of signed asynchronies reflect higher finger-tapping precision.

In line with our previous studies (Pecenka and Keller, 2009a,b), about two-thirds of the participants (13 out of 18) tended to predict (ratio > 1) the timing variations during finger tapping only (Tap), while the remaining individuals displayed a stronger tendency to track the ongoing tempo changes. In the more demanding dual-task conditions (Tap1B and Tap2B), the number of participants that showed behavioral evidence for relatively strong prediction decreased systematically (9 and 6 out of 18 participants, respectively). Overall, prediction/tracking ratios were highly correlated across the different finger-tapping conditions [Tap–Tap1B: *r* = 0.85; Tap–Tap2B: *r* = 0.74; Tap1B–Tap2B: *r* = 0.83; *p*s < 0.001].

In addition, a 3 (working-memory condition) × 2 (CC index) ANOVA was conducted the test whether the experimental manipulation had a different effect on the prediction (lag-0) and tracking indices (lag-1), which constitute the prediction/tracking ratio. This ANOVA yielded a significant effect of working-memory condition [*F*_(1.4, 23.0)_ = 5.81, *p* < 0.05, η^2^ = 0.26] and an interaction between working-memory condition and the two CC indices [*F*_(2, 34)_ = 11.61, *p* < 0.001, η^2^ = 0.41; see also Figure 2B]. Separate ANOVAs revealed that the experimental manipulation only had an effect on the lag-0 CCs [*F*_(2, 34)_ = 19.08, *p* < 0.001, η^2^ = 0.53] but not on the lag-1 CCs [*F*_(2, 34)_ = 0.65, *p* = 0.47, η^2^ = 0.04]. *Post-hoc* paired comparisons between the separate working-memory conditions revealed that lag-0 CCs were significantly lower (i.e., reflecting stronger prediction, due to the AC correction) during finger tapping without working-memory demands (Tap) than in the Tap1B [*t*_(17)_ = −3.54, *p* < 0.01] and Tap2B conditions [*t*_(17)_ = −6.27, *p* < 0.001]. As for the prediction/tracking ratio, lag-0 CCs did not differ significantly between the two working-memory conditions [*t*_(17)_ = −2.20, *p* = 0.04; the significance threshold is *p* = 0.016].

Additional ANOVAs were conducted to test whether the experimental manipulation had an effect on our other two measures of SMS performance: SMS accuracy (i.e., mean absolute asynchronies) and SMS precision (i.e., *SD* of signed mean asynchronies). Regarding SMS accuracy, a significant effect of the experiment manipulation was observed [*F*_(1.2, 20.6)_ = 7.25, *p* < 0.05, η^2^ = 0.30]. Local comparisons revealed that mean absolute asynchrony was lower for the tapping only (Tap) condition than for the dual-task conditions Tap1B [*t*_(17)_ = −2.74, *p* = 0.014] and Tap2B [*t*_(17)_ = −2.81, *p* = 0.012; the significance threshold due to Bonferroni correction is *p* = 0.016]. Differences in SMS accuracy between Tap1B and Tap2B were not statistically significant [*t*_(17)_ = −1.56, *p* = 0.14]. With respect to SMS precision a significant effect of the *n*-back manipulation on *SD* of signed asynchronies was also found [*F*_(2, 34)_ = 35.04, *p* < 0.001, η^2 = 0.67].^ Post-hoc *t*-tests revealed that finger-tapping precision was significantly higher in the tapping only condition (Tap) compared to Tap1B [*t*_(17)_ = −5.27, *p* < 0.001] and Tap2B [*t*_(17)_ = −8.37, *p* < 0.001]. Differences between Tap1B and Tap2B were not statistically significant for SMS precision [*t*_(17)_ = −1.98, *p* = 0.06]. Mean prediction/tracking ratios were significantly correlated with tapping accuracy [*r*_(16)_ = −0.48, *p* < 0.05] and precision [*r*_(16)_ = −0.75, *p* < 0.001]. That is, participants who displayed higher degrees of prediction during SMS with the tempo-changing pacing signals also synchronized their finger tapping with higher accuracy and precision.

#### Task difficulty

The three finger-tapping conditions (Tap, Tap1B, Tap2B) differed significantly regarding perceived task difficulty [*F*_(2, 34)_ = 266.83; *p* < 0.001; η^2^ = 0.94]. Subjectively perceived difficulty increased significantly with increasing working-memory demands from tapping only (Tap) over Tap1B to Tap2B [*t*_(17)_ < −8.63, *p*s < 0.001]. Furthermore, 2-back comparisons without finger tapping (2B) were perceived as significantly less difficult than Tap2B [*t*_(17)_ = 2.90, *p* < 0.01].

Regarding working-memory task performance, participants made significantly less *n*-back errors in the Tap1B condition compared to the Tap2B [*t*_(17)_ = −7.28; *p* < 0.001] and 2B condition [*t*_(17)_ = −5.82; *p* < 0.001], while performance in the two 2-back conditions (with and without finger tapping) was not significantly different [*t*_(17)_ = 0.37; *p* = 0.72].

### fMRI data

#### Positive correlates of temporal prediction during SMS

**Figure 3:**
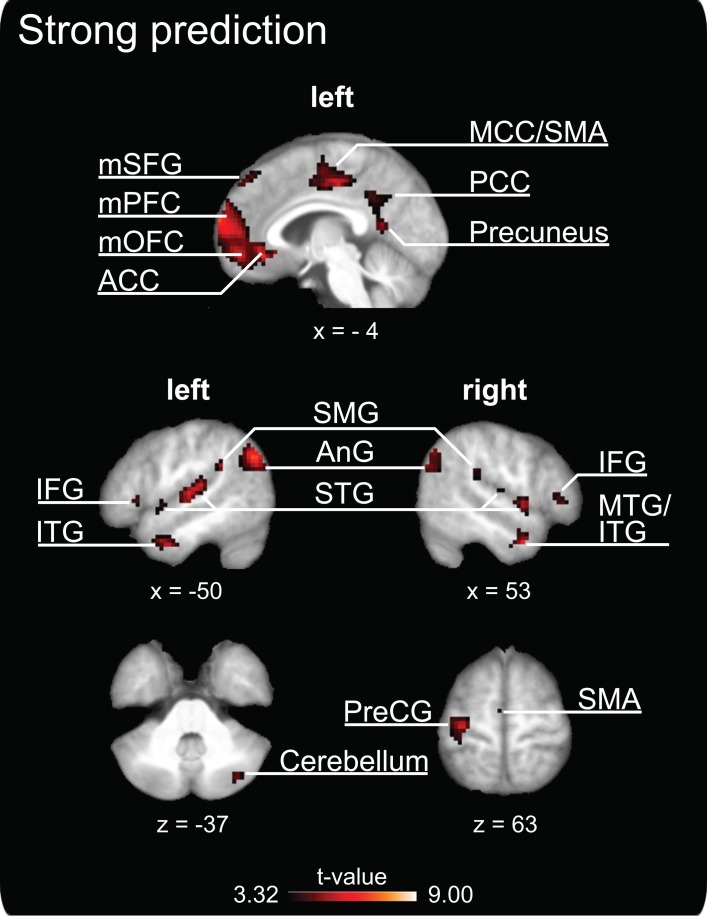
**Clusters of increased brain activity that covary positively with the degree of prediction during sensorimotor synchronization.** The depicted activations are significant at a threshold of *p* < 0.05, FDR corrected for multiple comparisons. Results are displayed according to neurological convention. The coordinates are given according to MNI space and activations are plotted on the mean anatomy of 18 participants. mSFG, medial superior frontal gyrus; mPFC, medial prefrontal cortex; mOFC, medial orbitofrontal cortex; ACC, anterior cingulate cortex; MCC, middle cingulate cortex; SMA, supplementary motor area; PCC, posterior cingulate cortex; IFG, inferior frontal gyrus; ITG, inferior temporal gyrus; SMG, supramarginal gyrus; AnG, angular gyrus; STG, superior temporal gyrus; MTG, middle temporal gyrus; PreCG, precentral gyrus.

The parametric analysis revealed that brain activation in a distributed network of areas covaried *positively* with the degree of prediction measured during SMS with a pacing signal that changed its tempo gradually (see Table 2 and Figure 3, all activations are significant at *p* < 0.05, FDR corrected). Large clusters of activation were revealed in medial cortical areas, including orbitofrontal and medial prefrontal cortex (Brodmann area, BA 10 and BA 11) extending into anterior cingulate cortex (ACC, BA 32), as well as in medial superior frontal gyrus (BA 8 and BA 9), middle cingulate cortex (BA 24 and BA 31) and SMA proper (BA 6), posterior cingulate cortex (BA 31) and precuneus. Bilateral activity clusters were revealed in temporal cortices covering the primary and secondary auditory cortex (BA 41, BA 42, and BA 22). In the left hemisphere, activation of the auditory cortex extended into the adjacent superior temporal gyrus (STG, BA 22) and middle temporal gyrus (BA 21). In the right hemisphere, the activation of the auditory cortex extended into the right posterior insular cortex. Additionally, bilateral activity was observed in the middle temporal gyrus extending into the inferior temporal gyrus and in the right hemisphere into the inferior frontal gyrus (IFG, pars orbitalis). Furthermore, activation clusters in the bilateral inferior parietal lobe (IPL, angular and supramarginal gyri, BA 39 and BA 40), right posterior middle temporal gyrus, left insular cortex, bilateral IFG (with a cluster on pars triangularis, BA 45, in the right hemisphere and two clusters in the left hemisphere on pars orbitalis and pars triangularis, BA 44), unilateral activity in left pre- and postcentral gyrus [primary motor areas, BA 4, dorsal premotor cortex (PMd), BA 6], left supramarginal gyrus (BA 1) and the right cerebellum (lobule VIIa/CrusI) also covaried with the degree of prediction.

**Table 2:** **Peak voxels of brain areas that covary positively with the degree of prediction during sensorimotor synchronization**.

			**Peak MNI coordinates**				**Prediction-2B**	
**Anatomical region**	**Hemisphere**	***k***	***x***	***y***	***z***	***Z***	***k***	***Z***
**MEDIAL REGIONS**								
Medial orbitofrontal cortex	R	825	3	35	−11	5.00	1061	5.43
Medial prefrontal cortex	L		−3	68	16	4.74		5.00
Anterior cingulate cortex	R		3	35	−8	4.42		4.91
Middle cingulate cortex	R/L	177	0	−19	43	4.37	510	4.79
SMA proper	R/L		0	−13	49	3.75		4.12
Precuneus	L	91	−3	−55	13	3.79	^a^	4.20
Middle cingulate cortex	L		−3	−46	34	3.35	^a^	3.76
Posterior cingulate cortex	R/L		0	−49	22	3.35	^a^	3.75
Medial superior frontal gyrus	R/L	48	0	50	43	3.72	107	4.01
**TEMPORAL REGIONS**								
Superior temporal gyrus	L	233	−54	−19	7	4.38	324^b^	4.24
Middle temporal gyrus	L		−66	−28	−2	3.54		3.37
Insular cortex	R	113	48	2	−2	5.16	146	5.33
Superior temporal gyrus	R		54	2	−2	3.91		3.95
Middle temporal gyrus	R	92	54	2	−29	4.33	157	4.47
IFG (pars orbitalis)	R		42	35	−17	3.73		3.80
Middle temporal gyrus	L	59	−48	5	−29	4.31	124	4.62
Inferior temporal gyrus	L		−51	−4	−32	4.08		4.43
Insular cortex	L	19	−45	2	−5	3.87	40	4.38
Middle temporal gyrus	R	18	69	−43	10	4.02	16	3.93
**PARIETAL REGIONS**								
Angular gyrus	L	149	−48	−73	37	4.85	197	5.18^c^
Supramarginal gyrus	R	62	66	−37	31	3.85	^d^	4.01
Angular gyrus	R	41	54	−70	28	3.68	70	3.98^e^
Supramarginal gyrus	R	44	66	−19	19	3.78	159	4.07
Supramarginal gyrus (BA 1)	L	15	−60	−19	40	3.48	^f^	3.66
**FRONTAL REGIONS**								
Precentral gyrus (BA 6)	L	122	−33	−22	67	4.64	189	5.23
IFG pars triangularis (BA 45)	R	21	51	32	1	3.57	36	3.71
IFG (pars orbitalis)	L	18	−36	20	−20	3.72	41	3.90^g^
IFG pars triangularis (BA 44)	L	17	−54	20	1	3.37	27	3.54
**CEREBELLUM**								
Lobule VIIa/Crus I	R	18	36	−76	−38	3.54	46	3.80
**AREAS ONLY FOR THE CONTRAST PREDICTION-2B**								
Middle temporal gyrus	L		−63	−55	−2		14	3.89
Cerebellum	L		−30	−76	−38		14	3.38
Precuneus	L		−6	−46	58		11	3.13

For each activation cluster with a minimal size of 11 voxels the stereotaxic coordinates of the peak and subpeaks are given according to MNI space, along with Z-values (p < 0.05, FDR corrected for multiple comparisons), anatomical localization, and total cluster size k. Please note that cluster extended in several brain regions across different lobes. L, left; R, right; SMA, supplementary motor area; IFG, inferior frontal gyrus; BA, Brodmann area.

a Areas are connected to the cluster of 510 voxels on the middle cingulate cortex.

b Cluster of 324 voxels extends into the supramarginal gyrus: x = −60, y = −40, z = 34, Z = 3.74.

c Z-value belongs to the peak coordinate of the cluster, x = −48, y = −73, z = 34.

d Area is connected to the cluster of 159 voxels on the right supramarginal gyrus.

e Z-value belongs to the peak coordinate of the cluster, x = 57, y = −67, z = 25.

f Area is connected to the cluster of 324 voxels on the left superior temporal gyrus.

g Z-value belongs to the peak coordinate of the cluster, x = −36, y = 20, z = −23.

#### Negative correlates of temporal prediction during SMS

**Figure 4:**
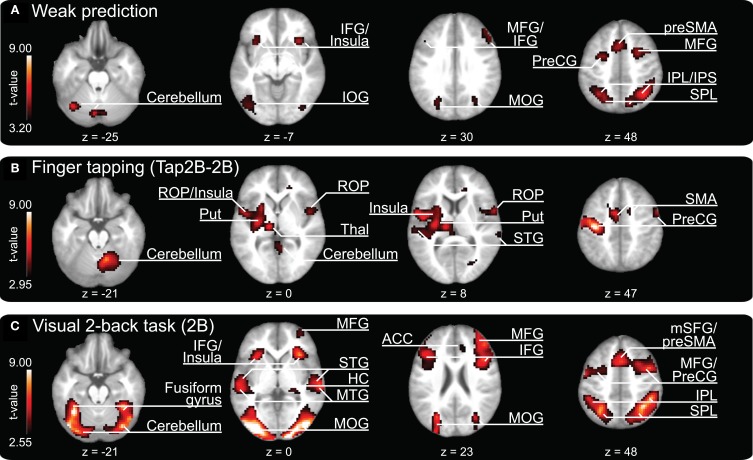
**Clusters of increased brain activity related to (A) weak temporal prediction (i.e., negative covariation with prediction/tracking ratios) and activation clusters revealed during (B) finger tapping (contrast “Tap2B-2B”) and (C) 2-back object comparisons and auditory perception of a pacing signal (2B).** The depicted activations are significant at a threshold of *p* < 0.05, FDR corrected. Results are displayed according to neurological convention. Color codes correspond to *t*-values as shown in each panel. The *z*-coordinates are given according to MNI space and activations are plotted on the mean anatomy of 18 participants. IFG, inferior frontal gyrus; IOG, inferior occipital gyrus; MFG, middle frontal gyrus; MOG, middle occipital gyrus; preSMA, pre-supplementary motor area; PreCG, precentral gyrus; IPL, inferior parietal lobe; IPS, intraparietal sulcus; SPL, superior parietal lobe; ROP, rolandic operculum; Put, putamen; Thal, thalamus; STG, superior temporal gyrus; SMA, supplementary motor area; HC, hippocampus; MTG, middle temporal gyrus; ACC, anterior cingulate cortex; mSFG, medial superior frontal gyrus.

Our parametric analysis also revealed areas that covaried *negatively* with prediction, i.e., these brain areas displayed higher activity when participants showed relatively weak prediction tendencies during SMS (see Figure 4A, and Table 3, all activations are significant at *p* < 0.05, FDR corrected). This network comprises clusters in the bilateral medial (vermis of lobule VI) and left lateral cerebellum (lobules VI and VIIa/CrusI/II), the left fusiform gyrus and inferior occipital lobe, the bilateral IPL extending into the superior parietal lobes and the right precuneus. Furthermore, the bilateral anterior insular cortex, the preSMA reaching anterior into the medial superior frontal gyrus, the bilateral frontal gyri (on the lateral surface anterior to the premotor area) and the right middle frontal gyrus reaching into BA 45 and the left IFG (BA 45) showed stronger activation when participants displayed weaker prediction tendencies in finger tapping.

**Table 3:** **Peak voxels of brain areas that covary negatively with the degree of prediction during sensorimotor synchronization**.

			**Peak MNI coordinates**			
**Anatomical region**	**Hemisphere**	***k***	***x***	***y***	***z***	***Z***
**OCCIPITAL, PARIETAL, AND CEREBELLAR REGIONS**						
Inferior parietal lobe	R	758	36	−58	52	4.68
Precuneus	R		15	−70	46	4.08
Inferior parietal lobe	L	551	−33	−58	49	4.03
Superior parietal lobe	L		−21	−64	46	3.95
Cereb. (Lob. VIIa/Crus I)	L	305	−39	−67	−23	4.43
Inferior occipital gyrus	L		−39	−76	−11	4.15
Cerebellum (Lob. VI)	L	122	−6	−76	−26	4.10
	R		9	−76	−23	3.50
Inferior occipital gyrus	R	41	39	−79	−8	3.33
**FRONTAL AND MEDIAL REGIONS, INSULAR CORTEX**						
Superior frontal gyrus	R	256	27	8	55	4.46
Pre-SMA	L/R	261	0	17	46	4.25
Insular cortex	R	137	30	23	1	4.80
Insular cortex	L	136	−33	17	−2	4.36
Middle frontal gyrus	L	137	−30	2	55	3.95
Middle frontal gyrus	R	124	51	32	31	3.59
Middle frontal gyrus	R	25	39	56	16	3.37
IFG p. triang. (BA 45)	L	19	−45	26	25	2.98

For each activation cluster with a minimal size of 11 voxels the stereotaxic coordinates of the peak and subpeaks are given according to MNI space, along with Z-values (p < 0.05, FDR corrected for multiple comparisons), anatomical localization, and total cluster size k. Please note that cluster extended in several brain regions across different lobes. L, left; R, right; Lob, lobule; Cereb., cerebellum; SMA, supplementary motor area; p.triang., pars triangularis; BA, Brodmann area.

#### Finger-tapping

The contrast “finger tapping and 2-back object comparisons vs. 2-back object comparisons without finger tapping (Tap2B-2B)” is informative about brain regions that are activated during SMS with a tempo-changing pacing signal. Coordinates of peak activations are listed in Table 4 and activation clusters are displayed in Figure 4B, all activations are significant at *p* < 0.05, FDR corrected. Consistent with previous observations on SMS with auditory signals, activation was exhibited bilaterally in temporal lobes covering the auditory cortex and in a network of distributed motor-related areas (for a review see Witt et al., 2008). In the left hemisphere, superior temporal lobe activation extended into operculum and insular cortex, as well as subcortically into left thalamus and putamen. A separate cluster of left-hemispheric activity was localized on the primary motor cortex (BA 4) extending into the PMd (BA 6) and somatosensory cortex (BA 1, BA 2, BA 3). In the right hemisphere, a large cluster of activation was revealed in the cerebellum (lobule VI), while smaller clusters were localized in rolandic operculum extending into insular cortex, STG, and precentral gyrus (BA 6). Finally, medial activation was exhibited bilaterally in the SMA (preSMA and SMA proper, BA 6) reaching into the middle cingulate cortex.

**Table 4:** **Peaks of increased brain activity during finger tapping (contrast “Tap2B-2B”)**.

			**Peak MNI coordinates**			
**Anatomical region**	**Hemisphere**	***k***	***x***	***y***	***z***	***Z***
**TEMPORAL REGIONS (EXTENDING INTO ADJACENT LOBES AND SUBCORTICAL STRUCTURES)**						
STG	L	1928	−54	−31	16	5.28
Parietal operculum	L		−42	−19	22	5.05
Thalamus	L		−12	−19	4	4.63
Putamen	L		−30	−16	7	4.42
Insular cortex	L		−39	−1	10	4.34
Rolandic operculum	R	174	60	5	10	4.13
Insular cortex	R		48	2	1	3.63
STG	R	77	66	−34	13	3.49
STG	R	15	42	−31	13	3.11
**FRONTAL REGIONS (EXTENDING INTO ADJACENT LOBES)**						
Postcentral g. (BA 4)	L	1636	−39	−19	49	5.70
SMA	L		−6	−7	61	5.24
Postcentral gyrus	L		−48	−13	43	5.00
MCC	L		−12	2	43	4.35
MCC	R		15	8	40	4.22
Precentral g. (PMd)	L		−45	−7	46	3.98
Caudate Nucleus	R		18	−4	22	3.10
Precentral gyrus	R	46	57	2	46	3.84
Orbitofrontal gyrus	R	32	15	44	−8	3.20
**CEREBELLUM**						
Lobule VI	R	717	12	−58	−17	5.23
Lobule VIIa/CrusI	L	15	−21	−88	−26	3.14

For each activation cluster with a minimal size of 11 voxels the stereotaxic coordinates of the peak and subpeaks are given according to MNI space, along with Z-values (p < 0.05, FDR corrected for multiple comparisons), anatomical localization, and total cluster size k. Please note that cluster extended in several brain regions across different lobes. L, left; R, right; STG, superior temporal gyrus; g., gyrus; BA, Brodmann area; SMA, supplementary motor area; MCC, middle cingulate cortex; PMd, dorsal premotor area.

#### 2-back object comparisons (2B)

The 2B experimental condition was introduced as a control condition, to reveal brain regions involved in working-memory computations (while listening to an auditory pacing signal, but without overt synchronization). Coordinates of peak activations are listed in Table 5 and activation clusters are displayed in Figure 4C, all activations are significant at *p* < 0.05, FDR corrected. In line with previous *n*-back studies requiring object identity comparisons (for an overview see Owen et al., 2005), activations were found in a bilateral frontoparietal network that spanned IFG (BA 44/45), middle frontal gyri, precentral gyrus and preSMA (BA 6), and the inferior parietal lobes. Furthermore, areas in bilateral temporal cortices and insular cortex were activated. The largest cluster of activations spanned bilateral occipital lobes, extending into parietal lobes, the fusiform gyrus, and cerebellum (lobules VI and VIIa/CrusI in both hemispheres).

**Table 5:** **Peaks of increased brain activity during 2-back object comparisons and auditory perception of a pacing signal (2B)**.

			**Peak MNI coordinates**			
**Anatomical region**	**Hemisphere**	***k***	***x***	***y***	***z***	***Z***
**OCCIPITAL, PARIETAL, AND CEREBELLAR REGIONS**						
Inferior occipital gyrus	R	6544	42	−79	−11	7.72
	L		−39	−70	−11	6.76
Middle occipital gyrus	L		−33	−91	4	6.87
	R		27	−91	4	6.14
Fusiform gyrus	L		−36	−82	−14	6.00
	R		36	−76	−20	5.27
Inferior parietal lobe	R		42	−46	43	5.50
	L		−30	−58	46	5.29
Superior parietal lobe	R		36	−58	55	5.29
	L		−30	−58	49	5.06
Cereb. (Lob. IV)	L		−36	−55	−23	5.70
Cereb. (Lob. VIIa/Crus I)	R		30	−79	−20	4.40
**FRONTAL REGIONS AND INSULAR CORTEX**						
Middle frontal gyrus	R	2677	45	5	55	5.48
Insular cortex	R		33	23	−2	5.31
Medial SFG	R/L		0	20	43	5.15
IFG (p. triang., BA 45)	R		57	23	28	5.00
MCC	R		9	32	31	4.29
Middle orbital gyrus	R		24	53	−14	3.92
Insular cortex	L	1258	−30	23	−5	5.38
Precentral gyrus	L		−42	2	31	4.47
IFG (p. triang., BA 45)	L		−45	23	22	4.30
Middle frontal gyrus	L		−39	56	16	3.74
**TEMPORAL REGIONS**						
MTG	L	405	−54	−31	4	5.08
STG	L		−36	−34	10	3.44
STG	R	331	69	−25	4	5.03
**SUBCORTICAL REGIONS**						
Thalamus	R	28	24	28	−2	3.04
Thalamus	R	17	9	−13	7	2.59

For each activation cluster with a minimal size of 11 voxels the stereotaxic coordinates of the peak and subpeaks are given according to MNI space, along with Z-values (p < 0.05, FDR corrected for multiple comparisons), anatomical localization, and total cluster size k. Please note that cluster extended in several brain regions across different lobes. L, left; R, right; Cereb., cerebellum; Lob., lobule; SFG, superior frontal gyrus; IFG, inferior frontal gyrus; p. triang., pars triangularis; BA, Brodmann area; MCC, middle cingulate cortex; MTG, middle temporal gyrus; STG, superior temporal gyrus.

#### Common and distinct activations during temporal prediction and 2-back object comparisons (2B)

A conjunction analysis that was conducted to identify neural regions that were commonly activated during both conditions revealed no significant activation clusters at *p* < 0.05, FDR corrected. In addition to this analysis, we calculated the contrast “prediction-2B” to explore further whether the brain network underlying temporal prediction was distinct from the activation pattern related to the visual working-memory task (compare Figures 3 and 4C). This analysis confirmed that all areas reported for prediction remain significant in that contrast (*p* < 0.05, FDR corrected), i.e., show stronger activation for prediction than in 2B (see last two columns of Table 2).

A second conjunction analysis was conducted in order to test whether there is overlap between the network *negatively* related to prediction and the visual working-memory task (2B). Results are listed in Table 6 (all significant at *p* < 0.05, FDR corrected) and confirm a highly overlapping network for both conditions.

**Table 6:** **Peak voxels of brain areas that covary negatively with the degree of prediction during sensorimotor synchronization *and* the 2B condition (conjunction analysis)**.

			**Peak MNI coordinates**			
**Anatomical region**	**Hemisphere**	***k***	***x***	***y***	***z***	***Z***
**OCCIPITAL, PARIETAL, TEMPORAL, AND CEREBELLAR REGIONS**						
Inferior parietal lobe	L	1264	−30	−58	46	4.77
Inferior occipital gyrus	L		−39	−70	−11	4.76
Cereb. (Lob. VIIa/Crus I)	L		−39	−67	−26	4.44
Superior occipital lobe	L		−24	−67	34	4.41
Fusiform gyrus	L		−36	−46	−20	3.14
Inferior parietal lobe	R	903	36	−55	52	5.52
Superior occipital lobe	R		30	−64	49	5.10
Cerebellum (Lob. VI)	L	102	−6	−76	−26	4.59
Inferior occipital gyrus	R	125	39	−79	−8	4.09
Cerebellum (Lob. VI)	R	29	9	−76	−23	3.56
ITG	R	19	48	−43	−14	3.13
Cerebellum (Lob. VI)	R	14	30	−61	−32	3.08
**FRONTAL AND MEDIAL REGIONS, INSULAR CORTEX**						
Superior frontal gyrus	R/L	611	0	20	43	4.93
Superior frontal gyrus	R		27	8	55	4.57
Middle frontal gyrus	R	286	45	32	31	3.97
Insular cortex	R	153	30	23	−2	5.15
Insular cortex	L	130	−30	23	−2	4.94
Middle frontal gyrus	L	104	−30	5	58	3.77
Inferior frontal gyrus	L	102	−42	26	25	3.54
Precentral gyrus	L	61	−39	2	31	3.49

For each activation cluster with a minimal size of 11 voxels the stereotaxic coordinates of the peak and subpeaks are given according to MNI space, along with Z-values (p < 0.05, FDR corrected for multiple comparisons), anatomical localization, and total cluster size k. Please note that cluster extended in several brain regions across different lobes. L, left; R, right; Cereb., cerebellum; Lob., lobule; ITG, inferior temporal gyrus.

## Discussion

The present fMRI study investigated the neural correlates of auditory temporal predictions during SMS with gradual tempo modulations. In our SMS task, participants tapped a finger in synchrony to an auditory pacing signal that contained tempo changes designed to resemble tempo modulations displayed in real performed music. Prediction ability during SMS was manipulated by means of a simultaneous visual *n*-back working-memory task comprising three levels of increasing difficulty: observation only (Tap), 1-back (Tap1B), and 2-back (Tap2B) object comparisons. Analyses of finger-tapping performance revealed that the experimental manipulation introduced by the simultaneous visual *n*-back task had a significant overall effect on the degree of prediction during SMS: prediction/tracking ratios decreased with increasing working-memory load. Additional analyses on the two components of the prediction/tracking ratio revealed that the working-memory manipulation significantly affected only the prediction index (i.e., the lag-0 CCs), while leaving the tracking index (i.e., the lag-1 CCs) unchanged. A parametric analysis conducted on the hemodynamic responses recorded by fMRI revealed that higher prediction/tracking ratios during SMS were *positively* related to stronger activation in a distributed network of cortico-cerebellar motor-related brain areas, medial cortical areas, and the auditory cortex together with adjacent temporal areas. In addition, *negative* covariation with prediction was found in brain areas that corresponded very closely to the frontoparietal network that was also activated during the working-memory task without finger tapping (condition 2B).

Our behavioral results confirmed that the experimental manipulation, which was introduced to increase cognitive load by applying a visual *n*-back task simultaneously with SMS, had the intended effect on finger-tapping performance: not only prediction/tracking ratios decreased with increasing working-memory load, but also synchronization accuracy and precision. The manipulation had a significant overall effect on all measures of SMS performance, but differences between the two single working-memory conditions (Tap1B and Tap2B) were statistically not significant. Nevertheless, the Tap2B condition was perceived as significantly more difficult and individuals made more errors in the 2-back working-memory task compared to the Tap1B condition. Altogether, our experimental manipulation proved effective for increasing variability in individual prediction/tracking ratios, which are typically highly stable under single-task conditions (see Pecenka and Keller, 2011). Thus, intraindividual variability in prediction/tracking ratios can be used to analyze the neural correlates of prediction tendencies during SMS. Consistent with our previous studies (Pecenka and Keller, 2009a,b), strong prediction tendencies during SMS were also significantly related to better synchronization performance (as indexed by higher SMS accuracy and precision).

### Positive correlates of temporal prediction during SMS

The parametric analysis addressing brain regions in which activation is positively related to prediction in SMS revealed a distributed cortico-cerebellar network that is traditionally associated with motor control, including such areas as dorsal premotor and motor cortices, SMA proper, IPL, and lateral cerebellum. These identified clusters correspond closely to the activation foci commonly reported in finger-tapping studies (see meta-analysis by Witt et al., 2008 for an overview). Our results suggest that the areas that we identified as being related to prediction may not only be important for motor control in general but may play a specific role in aspects of prediction related to motor timing and temporal adaptation during SMS. This assumption is consistent with previous studies: The PMd has not only been linked to simple and complex motor processes (e.g., Rao et al., 1993; Sadato et al., 1996; Hlustik et al., 2002; Ullen et al., 2003; Debaere et al., 2004) but has also been suggested to play a role in mediating auditory-motor interactions during finger tapping by extracting higher-order information from an auditory stimulus and integrating this information with motor processes so that appropriately timed actions can be executed (Chen et al., 2006, 2008a,b). SMA is also known to play a role in basic aspects of motor timing (e.g., Colebatch et al., 1991; Matelli et al, 1993; Rao et al., 1993; Ullen et al., 2003), but is also activated by perceptual timing tasks, independently of motor implementation (e.g., Macar et al., 2006; Bengtsson et al., 2009). In these studies, activation of SMA proper was positively related to sequence predictability (Bengtsson et al., 2009) and attention paid to time (Macar et al., 2006). Furthermore, the cerebellum is considered to be important for timing in motor, sensory, and cognitive domains (Ivry and Keele, 1989; Ivry et al., 2002; Bueti et al., 2008; O'Reilly et al., 2008) and it has been identified as playing a key role in the generation of internal forward and inverse models that support fine motoric timing (Wolpert et al., 1998; Ito, 2008). The posterior cerebellum, which was activated as part of the network related to prediction, has been linked to temporal processing underlying working-memory for rhythm (Jerde et al., 2011). Finally, the IPL (together with the premotor cortex) is part of a circuit involved in action-perception matching (e.g., Molenberghs et al., 2012) and is therefore important for the integration of sensory signals, for internal models and action execution (Miall, 2003), as well as for the perceptual prediction of upcoming events (Schubotz, 2007). Altogether, the present study adds to the current knowledge about these traditional motor areas by showing that they do not only play a role in explicit timing (cf. Coull et al., 2011; Witt et al., 2008), but may fulfill a specific function in situations that require temporal prediction during motor synchronization. However, because perceptual and motor processes are closely intertwined in the parametric design of our study, an assignment of separate components of the prediction-related brain network to specific processes pertaining to temporal prediction, sensorimotor integration, or motor timing and temporal adaptation must remain speculative.

Clusters in bilateral temporal cortices were also revealed to be part of the neural network related to prediction. In general, activation of auditory areas is not surprising as auditory stimulation (and scanner noise) was present during all conditions of the current experiment. However, it is remarkable that activation in the auditory cortex covaried with the degree of prediction even though auditory input was identical across all finger-tapping conditions. On the one hand, activation in auditory cortices may be related to (anticipatory) auditory imagery processes (Leaver et al., 2009; see Hubbard, 2010 for a review) that presumably support prediction during SMS (Keller, 2008). On the other hand, this modulation may reflect resources available for auditory processing, which varied depending on the cognitive load imposed by the working-memory dual task. Previous investigations using dual-task paradigms showed that working-memory load in one task reduces resources available for a second task, even when the two tasks draw upon different sensory modalities (e.g., De Fockert et al., 2001; Klemen et al., 2009). Thus, it has been suggested that working-memory load can impact upon attentional control, which, in turn influences perceptual (and post-perceptual) processes (Klemen et al., 2009). Studies on auditory attention have furthermore revealed that activity in primary and secondary auditory cortex is modulated by attention (e.g., Jäncke et al., 1999; Petkov et al., 2004). Regarding our dual-task paradigm, these observations are consistent with the idea that high visual working-memory load may have reduced the attentional resources available for auditory processing (and presumably also for sensorimotor integration and motor timing), as indicated by lower activation in STG during decreased prediction. Finally, variations in auditory cortical activation with differing degrees of prediction may reflect auditory-motor interactions during SMS. Previous studies on rhythm perception and production have reported functionally coupled neural activity in auditory and premotor cortex (Chen et al., 2006, 2008b), as well as SMA (Grahn and Rowe, 2009).

A further remarkable result is that clusters along the medial wall (medial prefrontal cortex and ACC, middle cingulate cortex, posterior cingulate cortex and precuneus) were found to be more activated when participants employed greater degrees of prediction. The medial prefrontal cortex (mPFC), together with ACC, is central to an array of higher cognitive functions including social-cognitive processes (e.g., Amodio and Frith, 2006; Gilbert et al., 2006; Frith, 2007; Carrington and Bailey, 2009) as well as processes underlying action control and monitoring, such as error detection and response conflict monitoring (e.g., Cohen et al., 2000; Ullsperger and von Cramon, 2004). Contemporary approaches that have attempted to identify a common function have suggested that mPFC and ACC subserve general performance monitoring, in the sense that expectations about action outcomes are formed and moment-by-moment discrepancies between actual and expected outcomes are detected (Alexander and Brown, 2010, 2011). Interestingly, a recent study revealed that mPFC/ACC play a role in predicting not only action outcomes (e.g., in terms of valence), but were also sensitive to violations of the *timing* of these expected outcomes in a two-choice prediction task (Forster and Brown, 2011). A different study that investigated the influence of temporal predictability of auditory rhythm sequences in a passive listening task reported that activation in bilateral middle frontal gyrus (BA 10; although slightly more lateral than our mPFC activation cluster) and medial motor areas (SMA and preSMA) covaried positively with the rhythm's predictability (Bengtsson et al., 2009). In line with these findings, our results are consistent with the hypothesis that enhanced activity in medial areas (and particularly in mPFC) could reflect the formation of precise sensory predictions of temporal sequences (cf. Bengtsson et al., 2009).

Furthermore, mPFC (among other areas) has been identified as one of the core regions in a network involved in making inferences about others' beliefs and predictions about other people's actions on the basis of their mental states (for reviews on mentalizing see Frith and Frith, 2003; Saxe, 2006; Frith, 2007; Carrington and Bailey, 2009). Specifically, mPFC was commonly activated when individuals were engaged in real-time social interactions with either a real human co-actor (McCabe et al., 2001; Decety et al., 2004), a virtual partner that behaved similar to a human (Schilbach et al., 2006; Fairhurst et al., 2013), or when they were led to believe that they were interacting with a real human actor as opposed to a computer (Gallagher et al., 2002; Ramnani and Miall, 2004). Remarkably, our medial prefrontal cortex cluster overlaps with or is in very close proximity to peak activations reported in all of the social interaction studies mentioned above. This result is consistent with the possibility that our participants may have effectively interpreted the computer-generated pacing signal in the SMS task as the action outcome of another human agent. This might appear surprising, as the auditory signal was rather artificial in comparison to the sounds that are normally produced during joint music making (i.e., the signal lacked variations in microtiming, pitch, timbre and intensity). However, the gradual tempo changes implemented in our pacing signal were deliberately designed to resemble changes in real performed music (i.e., ritardando and accelerando). Furthermore, studies on action simulation and theory of mind have demonstrated that even very reduced stimuli such as point-light figures (e.g., Dittrich et al., 1996; Sevdalis and Keller, 2011) and animated shapes (e.g., Heider and Simmel, 1944; Castelli et al., 2000) can convey social meaning, particularly when participants believed that the stimulus (e.g., the music they heard) was a human product (Steinbeis and Koelsch, 2009). In addition, the activation of brain areas related to such higher-level social-cognitive functions is in line with our previous finding that the degree of prediction during SMS with tempo-changing pacing sequences is positively correlated with self-reported musical perspective taking (i.e., strong agreement to the statement “To achieve successful ensemble coordination I pay attention to weaker musicians and adapt my playing accordingly”, see Pecenka and Keller, 2011). Taken together, our findings in combination with the above-mentioned evidence provide further support for the assumption, that music—even in a very reduced form—is not merely perceived as any auditory signal, but as a socially salient stimulus that conducts the presence of another agent whose intentions and actions can be interpreted and predicted (Molnar-Szakacs and Overy, 2006; Leman, 2007; Overy and Molnar-Szakacs, 2009).

Comparing the brain network underlying strong prediction with the activation pattern revealed during the visual working-memory task (2B), we have shown that the neural underpinnings of temporal prediction are distinct from the brain network implicated in working memory. This is not surprising, as strong prediction was possible mainly in the tapping only condition (Tap), wherein no working-memory task was performed. Thus, the absence of an overlap between the two networks does not imply that the two tasks did not interfere with each other, as this was clearly evident in the behavioral data. Specifically, we hypothesized that the introduction of a visual *n*-back task results in a decrease in the degree of prediction during SMS because both tasks draw on shared working-memory resources. With respect to our working-memory task, we have some indication that it may have drawn on a subvocal rehearsal process, instantiated in the phonological loop system of working memory (Baddeley, 2003). In order to interfere as little as possible with motor processes during finger tapping, the visual *n*-back task was designed to be nonverbal (as novel objects that do not have common names, the so-called “Fribbles”, were used). However, several of our participants reported having used a verbal strategy (by assigning names to the abstract objects) and subvocal rehearsal to facilitate *n*-back object comparisons. These reports are consistent with our finding that the neural working-memory network (associated with the 2B condition) included preSMA, an area that has been more typically observed during verbal compared to nonverbal working-memory tasks (and particularly identity comparisons; cf. Owen et al., 2005) and has been revealed as a key structure for subvocal rehearsal (e.g., Paulesu et al., 1993; Smith and Jonides, 1998; Baddeley, 2003). Therefore, interference between the two tasks in our dual-task paradigm may have occurred because both tasks draw on a shared process, namely subvocal rehearsal, instantiated in the phonological loop. The viability of this interpretation is furthermore supported by reports of interference effects of verbal processing tasks with internal time-keeping mechanisms during SMS (Sergent et al., 1993; cf. Rao et al., 1997). It has thus been suggested that internal timing during finger tapping may rely on the retrieval and rehearsal of internal auditory representations of time intervals (Rao et al., 1997; Witt et al., 2008), especially during conditions without an external pacing signal (e.g., continuation tapping). Finally, there are anecdotal reports that the learning of complex motor and rhythmic sequences (e.g., in drumming lessons) is facilitated by subvocal rehearsal.

### Negative correlates of temporal prediction during SMS

The parametric analysis on neural substrates related negatively to prediction tendencies during SMS revealed a close overlap with the frontoparietal network of brain areas activated during our visual working-memory condition (2B). The visual working-memory task was deliberately chosen to interfere with the degree of prediction during SMS and it resulted in lower prediction/tracking ratios in the Tap1B and Tap2B conditions compared to tapping only (Tap). Therefore, close overlap between brain areas that covary negatively with prediction and the brain network underlying the working-memory condition is plausible. This overlap, together with our finding that working-memory performance did not differ between the two 2-back conditions (Tap2B and 2B), is consistent with the assumption that individuals focused their attention (and presumably also working-memory resources) predominantly on the *n*-back task under dual-task conditions, while simultaneous finger tapping was performed rather automatically (and resulted in a decline in synchronization performance).

## Conclusion

In the current study, we have identified an extensive brain network related to prediction during SMS with auditory pacing sequences containing tempo changes that are similar to those that characterize expressive music performance. This network comprises motor-related cortico-cerebellar brain areas that mediate general motor control and, more specifically, perceptual prediction, sensorimotor integration, motor timing and temporal adaptation. Furthermore, our findings suggest that individuals may use higher-level cognitive functions related to mentalizing and perspective taking during action coordination with an auditory signal that bears resemblance to the action outcome of a human agent.

### Conflict of interest statement

The authors declare that the research was conducted in the absence of any commercial or financial relationships that could be construed as a potential conflict of interest.

## Footnotes

^1^The behavioral pilot study was conducted to ensure that our working-memory manipulation had the intended effect on individual prediction tendencies measured during SMS. The task and procedure were practically identical to the present fMRI study (see Material and Methods), except that participants were tested sitting upright in a quiet laboratory and trials did not include temporal jitter (this was only necessary for the fMRI design). Out of the 39 participants that were tested (mean age 26.1 years, 19 females), 34 participants displayed a tendency to predict rather than track the tempo changes (as evidenced by a prediction/tracking ratio >1) during finger tapping and object observation (Tap), while this was the case for only 24 participants during SMS and 1-back comparisons (Tap1B) and for 19 participants during SMS and 2-back comparisons (Tap2B). An analysis of variance yielded a significant main effect of experimental condition [*F*_(2, 76)_ = 71.36, *p* < 0.001, η^2^ = 0.65]. Paired comparisons between individual conditions revealed that prediction/tracking ratios decreased significantly from Tap over Tap1B to Tap2B [*t*_(38)_ > 3.84, *p*s < 0.001]. Twenty participants from this pilot sample volunteered to participate in the fMRI study about 6 months later.
